# Hsp90α Mediates BMI1 Expression in Breast Cancer Stem/Progenitor Cells through Facilitating Nuclear Translocation of c-Myc and EZH2

**DOI:** 10.3390/ijms18091986

**Published:** 2017-09-15

**Authors:** Yueh-Chun Lee, Wen-Wei Chang, Yi-Ying Chen, Yu-Hung Tsai, Ying-Hsiang Chou, Hsien-Chun Tseng, Hsin-Lin Chen, Chun-Chieh Wu, Ju Chang-Chien, Hsueh-Te Lee, Huei-Fan Yang, Bing-Yen Wang

**Affiliations:** 1 Department of Radiation Oncology, Chung Shan Medical University Hospital, Taichung 40201, Taiwan; lee.yuehchun@gmail.com (Y.-C.L.); hideka.chou@gmail.com (Y.-H.C.); rad.tseng@msa.hinet.net (H.-C.T.); cznpzz@gmail.com (H.-L.C.); kubernet332@gmail.com (C.-C.W.); benlinda.tw@yahoo.com.tw (H.-F.Y.); 2 Institute of Medicine, Chung Shan Medical University, Taichung 40201, Taiwan; 3 School of Biomedical Sciences, Chung Shan Medical University, Taichung 40201, Taiwan; changww@csmu.edu.tw (W.-W.C.); abc19950721@gmail.com (Y.-Y.C.); mapleleaves710301@hotmail.com (Y.-H.T.); swan1204@hotmail.com (J.C.-C.); 4 Department of Medical Research, Chung Shan Medical University Hospital, Taichung 40201, Taiwan; 5 Department of Medical Imaging and Radiological Sciences, Chung Shan Medical University, Taichung 40201, Taiwan; 6 School of Medicine, Chung Shan Medical University, Taichung 40201, Taiwan; 7 Institute of Biochemistry, Microbiology and Immunology, Chung Shan Medical University, Taichung 40201, Taiwan; 8 Institute of Anatomy and Cell Biology, School of Medicine, National Yang Ming University, Taipei 11529, Taiwan; incubator.lee@ym.edu.tw; 9 Department of Nursing, Chung shan Medical University Hospital, Taichung 40201, Taiwan; 10 Division of Thoracic Surgery, Department of Surgery, Changhua Christian Hospital, Changhua City 50006, Taiwan; 11 School of Medicine, Chung Shan Medical University, 40201 Taichung, Taiwan; 12 Institute of Genomics and Bioinformatics, National Chung Hsing University, Taichung 40201, Taiwan; 13 School of Medicine, College of Medicine, Kaohsiung Medical University, Kaohsiung 80708, Taiwan

**Keywords:** Hsp90α, BMI1, breast cancer stem cells, EZH2, c-Myc, nuclear translocation

## Abstract

Heat shock protein 90 (Hsp90) is a molecular chaperone that facilitates the correct folding and functionality of its client protein. Numerous Hsp90-client proteins are involved in cancer development. Thus, Hsp90 inhibitors have potential applications as anti-cancer drugs. We previously discovered that Hsp90α expression increased in breast cancer stem cells (BCSCs), which can initiate tumorigenesis and metastasis and resist treatment. In the present study, we further demonstrated that 17-dimethylaminoethylamino-17-demethoxygeldanamycin (17-DMAG), an inhibitor of Hsp90, could suppress the self-renewal of BCSCs by downregulating B lymphoma Mo-MLV insertion region 1 homolog (BMI1), a polycomb family member with oncogenic activity in breast cancer. Through immunoprecipitation analysis, we found that BMI1 did not interact with Hsp90α and that the downregulation of BMI1 by 17-DMAG was mediated by the inhibition of c-Myc and enhancement of zeste homolog 2 (EZH2) expression. The transcriptional and BMI1 promoter-binding activities of c-Myc in BCSCs were inhibited by 17-DMAG treatment. The overexpression of EZH2 attenuated the inhibitory effect of 17-DMAG on BMI1 and c-Myc expression. Furthermore, Hsp90α could be co-immunoprecipitated with c-Myc and EZH2 and bind to the BMI1 promoter. Treatment with 17-DMAG decreased the nuclear expression of EZH2 and c-Myc but not that of Hsp90α. In conclusion, our data suggested that Hsp90α could positively regulate the self-renewal of BCSCs by facilitating the nuclear translocation of c-Myc and EZH2 to maintain BMI1 expression.

## 1. Introduction

Breast cancer is the most common cancer and the leading cause of cancer-related death among females worldwide [1]. Breast cancer can be classified into several subtypes on the basis of the expression profiles of hormone receptors and human epidermal growth factor receptor 2 (HER2): luminal A, luminal B, HER2+, and triple negative/basal-like breast cancer [2]. Triple-negative breast cancer cells (TNBC) are associated with the poor prognosis of patients and lack effective chemotherapy or targeting therapy agents [3,4]. Cancer stem/progenitor cells (CSCs) are a subpopulation of cancer cells with tumor initiation capacity and have an important role in cancer drug resistance, relapse, and metastasis [5,6]. Breast cancer stem/progenitor cells (BCSCs) are breast cancer cells that are characterized by the expression of specific markers, such as CD24-CD44^+^ [7], and the activity of intracellular aldehyde dehydrogenase (ALDH) [8]; these cells can be enriched through tumorsphere cultivation [9,10]. BCSCs are resistant to therapy, including radiation treatment [11,12] or chemotherapy [13,14]. Thus, targeting BCSCs may be a potential therapeutic strategy against breast cancer [5,10]. B lymphoma Mo-MLV insertion region 1 homolog (BMI1) is a member of the polycomb repression complex 1 (PRC1) family and regulates the self-renewal of normal and malignant stem cells by preventing senescence and apoptosis [15,16]. BMI1 is an independent prognostic factor of TNBC [17] and is essential for the self-renewal of BCSCs [18].

Heat shock protein 90 (Hsp90) is a molecular chaperone, a cellular protein that facilitates and maintains the correct folding of its client protein through the binding of ATP molecule [19]. Many Hsp90-client proteins, such as protein kinases, steroid hormone receptors, cycle regulators, and telomerase, are involved in oncogenesis [20]. Therefore, Hsp90 inhibitors have potential applications as anti-cancer drugs. Several Hsp90 inhibitors, which exert their inhibitory effect by competitively binding at the ATP binding site of the Hsp90 dimer, have been developed, and their therapeutic effect on cancer have been examined in clinical trials [21]. We previously discovered that Hsp90α expression is upregulated in ALDH^+^ BCSCs [22]. Hsp90 inhibitors, including geldanamycin and its analog 17-dimethylaminoethylamino-17-demethoxygeldanamycin (17-DMAG), could decrease the population of ALDH^+^ BCSCs and the protein expression of Akt, a well-known client protein of Hsp90 that regulates cell survival and proliferation [22]. This finding suggested that Hsp90 inhibitors are potential and effective cancer chemotherapeutic drugs that target BCSCs. However, the mechanisms that underlie the anti-BCSC action of Hsp90 inhibitors should be further investigated.

In the present study, we further investigated the molecular mechanism that underlies the BCSC-targeting effect of 17-DMAG and discovered that BMI1 is downregulated in 17-DMAG-treated TNBC-derived mammosphere cells. We also demonstrated that the downregulation of BMI1 by 17-DMAG is mediated by the suppression of c-Myc transcriptional activity and the downregulation of the enhancer of zeste homolog 2 (EZH2), a PRC2 member. The overexpression of EZH2 prevents the downregulation of BMI1 or c-Myc in 17-DMAG-treated BCSCs. Finally, we discovered that Hsp90α complexes with EZH2 and c-Myc on the BMI1 promoter and functions as a shuttle that translocates EZH2 and c-Myc to the nucleus. Our data suggested that Hsp90α could positively regulate the self-renewal of BCSCs by facilitating the nuclear translocation of c-Myc and EZH2 to maintain BMI1 expression.

## 2. Results

### 2.1. 17-DMAG Inhibits the Self-Renewal Capability of BCSCs and Downregulates Their Expression of BMI1

**Figure 1:**
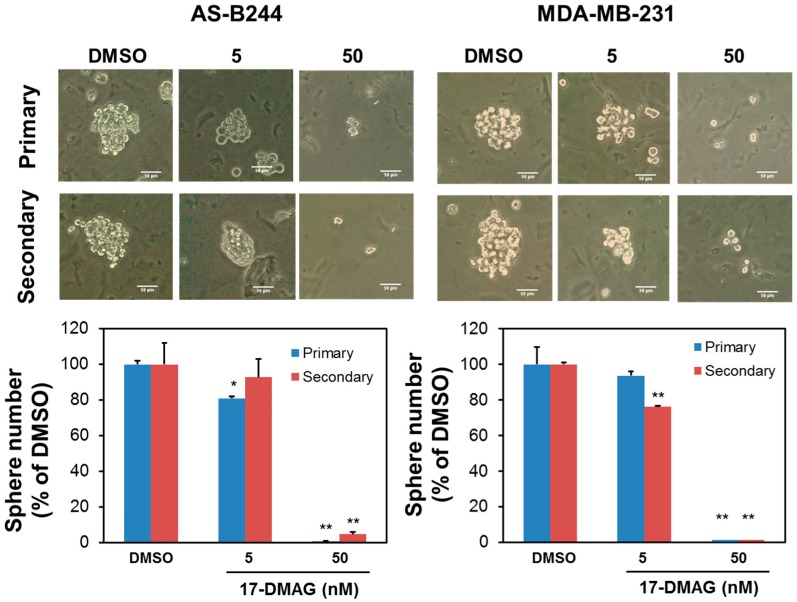
17-DMAG inhibited proliferation of mammospheres derived from Triple-negative breast cancer cells (TNBC). AS-B244 or MDA-MB-231 TNBC cells were cultured under mammosphere cultivation conditions and treated with 0.1% dimethyl sulfoxide (DMSO) or the indicated concentration of 17-DMAG. The number of primary mammospheres was counted at Day 7 and mammospheres were then collected, dissociated into single cells, and subjected to secondary mammosphere formation. Data were presented as the percentage of DMSO control. * *p* < 0.05; ** *p* < 0.01.

**Figure 2:**
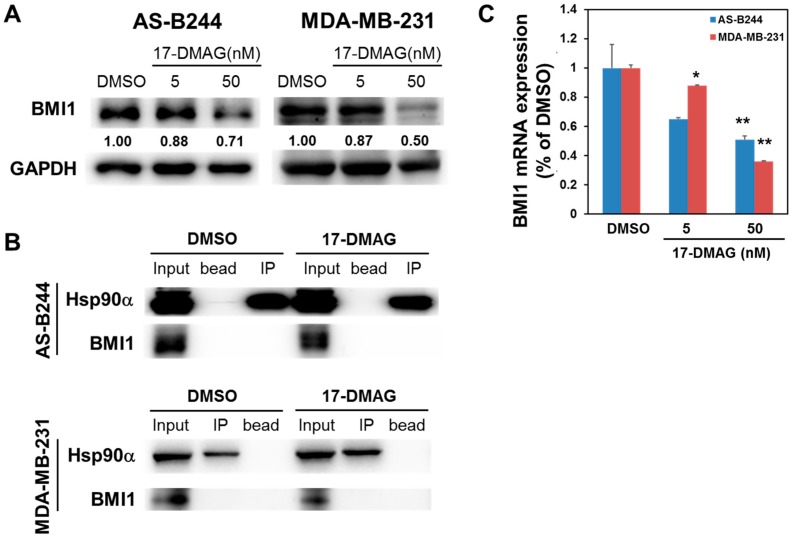
17-DMAG down-regulated B lymphoma Mo-MLV insertion region 1 homolog (BMI1) expression in TNBC mammospheres at mRNA and protein level and BMI1 was not interacted with Hsp90α. AS-B244 or MDA-MB-231 TNBC cells were performed mammosphere cultivation for 48 h and then treated with 0.1% DMSO or indicated concentration of 17-DMAG. The proteins were extracted at 96 h after treatment and used for determination of BMI1 protein expression with Western blot (**A**). Inserted numbers in (**A**) presented the relative expression level when compared to DMSO control. The binding of BMI1 and Hsp90α under 50nM 17-DMAG treatment was determined by immunoprecipitation with anti-Hsp90α antibody (**B**). The total RNA were isolated at 48 h after treatment and BMI1 mRNA expression was measured by SYBR Green-based qRT-PCR (**C**). * *p* < 0.05; ** *p* < 0.01.

We previously demonstrated that 17-DMAG could inhibit the expression of ALDH^+^ in AS-B244 human breast cancer cells [22]. We enriched BCSCs through mammosphere cultivation to further examine the effect of 17-DMAG on the self-renewal of BCSCs [10]. As shown in Figure 1, 17-DMAG inhibited the mammosphere formation of AS-B244 and MDA-MB-231 TNBC cells in a dose-dependent manner. This finding suggested that 17-DMAG suppresses the proliferation of TNBC CSCs under non-adherent and serum-free cultivation conditions. Several studies have reported that BMI1 positively regulates the self-renewal capability and tumorigenicity of BCSCs [23,24,25,26]. We next examined the effect of 17-DMAG on BMI1 expression in AS-B244 or MDA-MB-231 mammosphere cells. Our Western blot data indicated that 17-DMAG inhibited BMI1 expression in AS-B244 and MDA-MB-231 mammosphere cells (Figure 2A). Then, we used the immunoprecipitation method to determine if BMI1 is a client protein of Hsp90α. We found that the BMI1 protein is not co-precipitated by the anti-Hsp90α antibody (Figure 2B). Through quantitative RT-PCR, we observed that BMI1 mRNA expression in AS-B244 and MDA-MB-231 mammosphere cells are downregulated by 17-DMAG treatment in a dose-dependent manner (Figure 2C). These results indicated that 17-DMAG suppresses BMI1 expression through a transcriptional regulatory mechanism.

### 2.2. Inhibitory Effect of 17-DMAG on BMI1 Expression Is Associated with Downregulated c-Myc Transcriptional Activity

**Figure 3:**
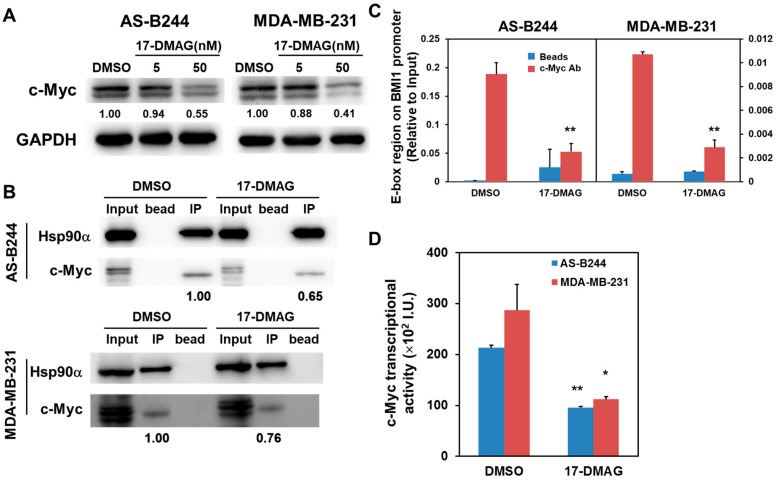
The transcriptional activity of c-Myc in TNBC mammospheres was suppressed by 17-DMAG. The mammospheres were cultured from AS-B244 or MDA-MB-231 TNBC cells for 96 h under the treatment of 0.1% DMSO or 17-DMAG (5 or 50 nM in (**A**) and 50 nM for (**B**). The protein expression of c-Myc was determined by Western blot (**A**) and the interaction between c-Myc and Hsp90α was determined by immunoprecipitation of anti-Hsp90α antidody (**B**). The binding of c-Myc to the E-box (-CACGTG-) region of the BMI1 promoter was determined by chromatin immunoprecipitation with anti-c-Myc antibody and the qPCR method. Data were presented as the relative expression level to the input chromatin of each sample (**C**). Transcriptional activity of c-Myc in TNBC mammospheres under 50nM 17-DMAG treatment was determined with a luciferase-based reporter assay (**D**). * *p* < 0.05; ** *p* < 0.01.

c-Myc is a transcriptional factor that controls BMI1 expression [27]. Western blot results indicated that 17-DMAG inhibits c-Myc expression in a dose-dependent manner (Figure 3A). The physical interaction between c-Myc and Hsp90 in Ras-overexpressed MCF7 breast cancer cells [28]. Subsequently, we examined the effect of 17-DMAG on the interaction between Hsp90α and c-Myc and found that 17-DMAG decreases the binding of Hsp90α and c-Myc in AS-B244 and MDA-MB-231 mammosphere cells (Figure 3B). Using chromatin immunoprecipitation (ChIP) analysis, we found that 17-DMAG treatment inhibits the binding of c-Myc on the E-box region in the BMI1 promoter (Figure 3C). Furthermore, the results of a luciferase-based reporter assay demonstrated that 17-DMAG downregulates the transcriptional activity of c-Myc in AS-B244 and MDA-MB-231 mammosphere cells (Figure 3D). These results indicated that the loss of c-Myc protein due to 17-DMAG mediated inhibition of Hsp90α resulted in decreased transcription of c-Myc target genes, including BMI1 in BCSCs.

### 2.3. 17-DMAG-Idnuced Inhibition of BMI1 and c-Myc Expression in BCSCs Is Associated with Downregulated EZH2 Expression

**Figure 4:**
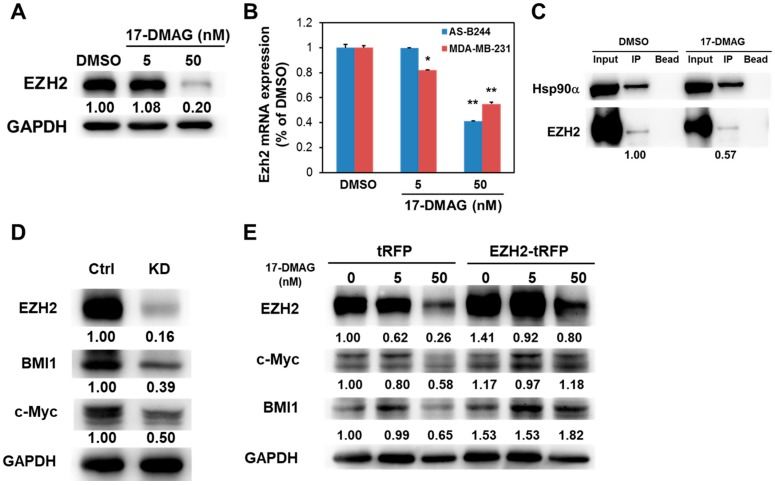
EZH2 expression in MDA-MB-231 mammospheres was suppressed by 17-DMAG. (**A**,**B**) The protein (**A**) or mRNA (**B**) expression of EZH2 in MDA-MB-231 mammospheres after 17-DMAG treatment (5 or 50 nM) was determined at 96 h (**A**) or 48 h (**B**). * *p* < 0.05; ** *p* < 0.01. (**C**) The interaction between EZH2 and Hsp90α in mammospheres of MDA-MB-231 after 17-DMAG treatment (50 nM) was determined by immunoprecipitation with anti-Hsp90α antibody; (**D**) Knockdown of EZH2 in primary mammosphere from MDA-MB-231 cells was performed by lentiviral delivery of LacZ- (Ctrl) or EZH2- (KD) specific shRNAs and total proteins were extracted for determination of EZH2, BMI1 or c-Myc expression by Western blot; (**E**) Overexpression of EZH2 was performed by lentiviral delivery of tRFP or EZH2 gene into MDA-MB-231 cells and selected by 20 µg/mL BSD for five days. Successfully-transduced cells were then cultured into mammosphere, treated with 0.1% DMSO or 17-DMAG (5 or 50 nM) for 96 h, and extracted the total proteins for determination of BMI1, c-Myc, EZH2, or GAPDH expression by Western blot. Inserted numbers indicated relative protein expression levels in comparison with the control.

EZH2, a member of PRC2, can transactivate c-Myc expression in glioblastoma cancer stem cells [29]. We tested whether EZH2 plays a role in the downregulation of BMI1 and c-Myc expression under 17-DMAG treatment. 17-DMAG inhibited EZH2 expression in MDA-MB-231 mammosphere cells at the protein (Figure 4A) and mRNA (Figure 4B) levels in a dose-dependent manner. A recent work has demonstrated that EZH2 directly interacted with Hsp90α within murine T cells, and the inhibition of Hsp90α by AYU922 induced rapid degradation of EZH2 [30]. We also observed that EZH2 interacts with Hsp90α in MDA-MB-231 mammosphere cells, and 17-DMAG can attenuate this interaction (Figure 4C). Through RNA interference technique, we further found that BMI1 and c-Myc expression decreases in MDA-MB-231 mammosphere cells after EZH2 knockdown (Figure 4D). We overexpressed EZH2 in MDA-MB-231 cells to further validate the involvement of EZH2 in the 17-DMAG-mediated downregulation of BMI1 and c-Myc. We performed Western blot analysis to observe BMI1 or c-Myc expression after 17-DMAG treatment. As shown in Figure 4E, EZH2 overexpression diminished the inhibitory effect of 17-DMAG on BMI1 and c-Myc expression.

### 2.4. 17-DMAG Interferes with the Hsp90α-Facilitated Nuclear Translocation of c-Myc and EZH2

**Figure 5:**
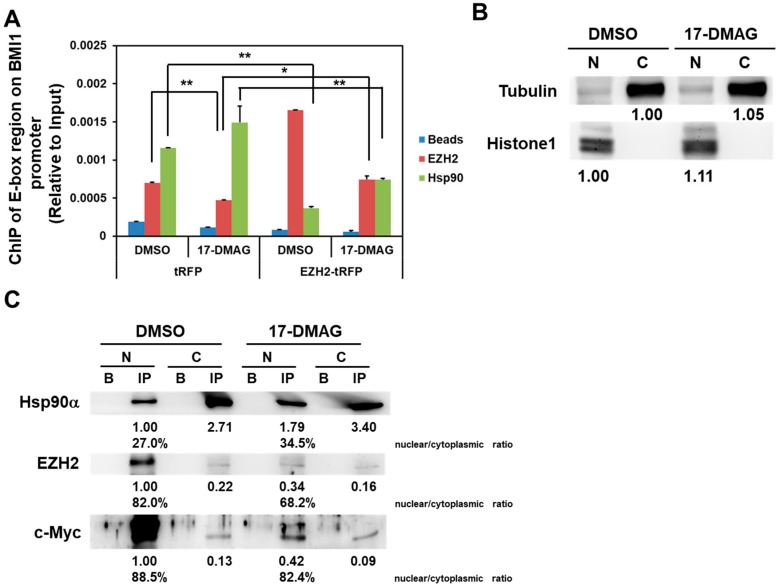
Hsp90α facilitated the nuclear translocation of c-Myc and EZH2 to BMI1 promoter. (**A**) The binding of EZH2 or Hsp90α on the E-Box region of BMI1 promoter in MDA-MB-231 mammospheres was determined by the ChIP method with anti-EZH2 or anti-Hsp90α antibody, respectively. Data were presented as the relative expression level to input chromatin of each sample. * *p* < 0.05; ** *p* < 0.01; (**B**) The purity of the cytoplasmic or nuclear fraction from MDA-MB-231 mammospheres was confirmed by Western blot analysis of tubulin (cytoplasmic specific protein) or histone 1 (nuclei specific protein). An equal amount of nuclear or cytoplasmic proteins from DMSO- or 17-DMAG-treated MDA-MB-231 mammospheres was used for immunoprecipitation analysis; (**C**) The contents of Hsp90/ EZH2/c-Myc complex in cytoplasmic or nuclear fraction proteins of MDA-MB-231 mammospheres were determined by immunoprecipitation with anti-Hsp90α antibody and analyzed by Western blot. N, nuclear; C, cytoplasmic; B, beads only; IP, immunoprecipitation.

We investigated the possibility of the formation of the Hsp90α/c-Myc complex within the BMI1 promoter. Using anti-Hsp90α or anti-EZH2 antibody to perform ChIP analysis, we amplified the c-Myc binding region within the BMI1 promoter through qRT-PCR. The results indicated that Hsp90α or EZH2 complexes with c-Myc to bind with the BMI1 promoter (Figure 5A, DMSO group of tRFP overexpressed cells). Treatment with 17-DMAG decreases the binding of EZH2/c-Myc complex on the BMI1 promoter, whereas the overexpression of EZH2 can overcome the inhibitory effect of 17-DMAG (Figure 5A). Given that Hsp90α participates in the nuclear–cytoplasmic shuttling of the mineralocorticoid receptor [31], we then investigated the effect of 17-DMAG on the Hsp90α-mediated nuclear–cytoplasmic shuttling of EZH2 or c-Myc. We isolated nuclear and cytosolic proteins and used equal amounts of nuclear and cytoplasmic proteins for immunoprecipitation (Figure 5B). When anti-Hsp90α antibody was used to perform immunoprecipitation, the Hsp90α/c-Myc/EZH2 complex localized in the nucleus (Figure 5C, DMSO group). By calculating the nuclear/cytoplasmic ratio of Hsp90α-shuttled EZH2- or c-Myc, we found that only EZH2 content decreased from 82.0% to 68.2% in response to 17-DMAG treatment (Figure 5C. However, 17-DMAG treatment decreased the nuclear content of Hsp90α-shuttled EZH2 and c-Myc (Figure 5C, 17-DMAG group), but not the nuclear entrance of Hsp90α (Figure 5C). These results suggested that the inhibition of Hsp90α activity by 17-DMAG leads to the decrease of the nuclear translocation of EZH2 and c-Myc and causes the downregulation of BMI1 in BCSCs.

## 3. Discussion

BMI1, a PRC1 member, prevents cellular senescence through its transcriptional repressor function, thus maintaining the self-renewal of normal or malignant stem cells [32]. Targeting BMI1 expression by PTC596, a novel small molecule inhibitor of BMI1, exhibits effective anti-CSC activity in animal models without interfering with normal stem cells [33]. In the present study we discovered, for the first time, that 17-DMAG, an Hsp90 inhibitor, downregulates BMI1 in TNBC CSCs. 17-DMAG displays anti-tumor activity in MDA-MB-231 xenograft SCID mice model [34] and is retained longer in tumors than in normal tissues [35]. A phase I clinical trial has been conducted to determine the toxicity of 17-DMAG in patients with advanced malignancies, including stage IV and recurrent breast cancers; the results of the trial revealed that the patients tolerated twice-weekly intravenous infusions of 17-DMAG well [36]. Our results, and those of previous studies, collectively suggested that 17-DMAG is a potential chemotherapeutic drug for breast cancer.

**Figure 6:**
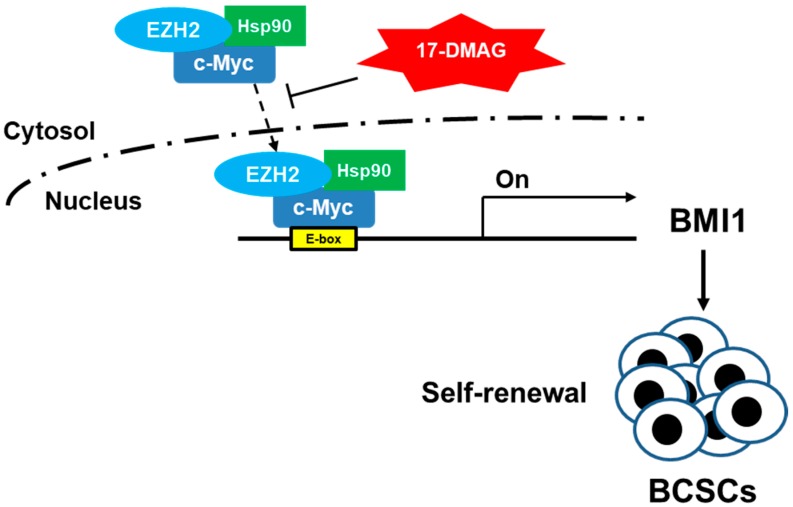
The working hypothesis of Hsp90-mediated BMI1 expression in BCSCs through the EZH2 and c-Myc pathways. One of the functions of Hsp90α is to facilitate the nuclear translocation of EZH2 and c-Myc and binding to the E-Box region of the BMI1 promoter to drive BMI1 expression and regulates the self-renewal of BCSCs. The Hsp90α mediated BMI1 expression could be inhibited by 17-DMAG, a specific Hsp90 inhibitor, which binds to ATP-binding pocket of the Hsp90 dimer.

BMI1, EZH2, and c-Myc have a complicated relationship. The expression of c-Myc-accelerated BMI1/estrogen receptor in human mammary epithelial cells induces tumor formation [37]. In breast cancer cells, BMI1 can repress the dickkopf WNT signaling pathway inhibitor 1 and induce c-Myc expression to further activate BMI1 expression [38]. c-Myc and EZH2 interact in glioblastoma CSCs, and c-Myc overexpression rescues the inhibitory effect of 3-deazaneplanocin A, an EZH2 inhibitor, on targeted glioblastoma CSCs [29]. Moreover, silencing c-Myc in embryonic stem cells (ESCs) suppresses the expression of PRC2 proteins and upregulates the expression of genes involved in primitive endoderm differentiation [39]. This finding suggested that the undifferentiated status of ESCs is maintained by the interaction between c-Myc and PRC2. In the present study we discovered, for the first time, that BMI1, c-Myc, and EZH2 are linked by Hsp90α. 17-DMAG treatment decreases c-Myc and EZH2 content in the nuclei of TNBC CSCs (Figure 5C) and the binding of EZH2 on the E-box region of the BMI1 promoter (Figure 5B) without interfering with the nuclear translocation of Hsp90α (Figure 5C). These results suggested that Hsp90α could function as a shuttle that facilitates the nuclear transport of EZH2 and c-Myc to mediate BMI1 transcription in TNBC CSCs and lead to the self-renewal of these cells (Figure 6). The central region of Hsp90α involved in shuttling EZH2 and c-Myc from the cytoplasm to the nucleus requires further study.

## 4. Materials and Methods

### 4.1. Cell Culture and Reagents

MDA-MB-231 was purchased from The Bioresource Collection and Research Center in Taiwan (Hsinchu, Taiwan) and was maintained in DMEM medium containing 10% fetal bovine serum (FBS). AS-B244 was obtained from Alice L. Yu in Institute of Stem Cell and Translational Cancer Research, Chang Gung Memorial Hospital at Linkou and Chang Gung University (Taoyuan, Taiwan) and was maintained in MEMα medium containing 10% FBS and 5 µg/mL insulin (Sigma-Aldrich, St. Louis, MO, USA). 17-DMAG was purchased from LC Laboratories (Woburn, MA, USA) and dissolved in dimethyl sulfoxide (DMSO) as a stock of 50 mM.

### 4.2. Mammosphere Cultivation

AS-B244 or MDA-MB-231 cells were suspended in DMEM/F12 medium containing 0.4% BSA (Sigma-Aldrich), 20 ng/mL epidermal growth factor (PeproTech, Rocky Hill, NJ, USA), 20 ng/mL basic fibroblast growth factor (PeproTech), 4 µg/mL heparin (Sigma-Aldrich), 1 µg/mL hydrocortisone (Sigma-Aldrich), and 5 µg/mL insulin (Sigma-Aldrich) as 1000 cells/mL. The number of primary mammospheres was counted at Day 7 and collected by 100 µm cell strainer (BD Biosciences, Franklin Lakes, NJ, USA) for secondary mammosphere culture after dissociation into single cell suspension with HyQTase treatment (HyClone Laboratories, Logan, UT, USA)

### 4.3. Western Blot

Cells were lysed with NP-40 lysis buffer and 25 µg of total protein were separated by SDS-PAGE and transferred to 0.22 µm polyvinylidene fluoride (PVDF) membranes (Pall Corporation, New York, NY, USA). After blocking with 5% skimmed milk in Tris-buffered saline (TBS) containing 0.05% Tween-20 (TBS-T), membranes were incubated with primary antibody in solution I of SignalBoost^TM^ Immunodetection Enhancer kit (Calbiochem, San Diego, CA, USA) at 4 °C overnight. After washing and secondary incubation in 0.5% skimmed milk/TBS-T, the signal was further developed by ECL-plus chemoluminescence substrate (Perkin-Elmer, Waltham, MA, USA) and captured with a FUSION Solo S Imaging system (Vilber Lourmat, MArne-la-Valée, France). Rabbit anti-human BMI1 polyclonal antibody was purchase form Novus Biologicals (Littleton, CO, USA). Rat anti- human Hsp90α monoclonal antibody was purchased from Enzo Life Sciences, Inc. (Farmingdale, NY, USA). Mouse anti-human EZH2 monoclonal antibody was purchased from BD Transduction Laboratories. Rabbit anti-human c-Myc antibody, rabbit anti-glyceraldehyde-3-phosphate dehydrogenase (GAPDH) polyclonal antibody, and mouse monoclonal anti-α tubulin antibody were purchased from GeneTex International Corporation (Hsinchu, Taiwan). Mouse monoclonal anti-histone H1 antibody was purchased from Santa Cruz Biotechnology, Inc. (Dallas, TX, USA).

### 4.4. Quantitative Real-Time RT-PCR

Total RNA were extracted by a Quick RNA MiniPrep kit (Zymo Research, Irvine, CA, USA) and reverse transcribed to cDNA using oligo(dT) primer (RevertAid First Strand cDNA Synthesis Kit, Fermentas). Ten nanograms (10 ng) of cDNA was used for gene detection by the SYBR Green-based quantitative real-time RT-PCR method, which was conducted on an ABI StepOnePlus^TM^ Real-Time PCR system and analyzed with the StepOne software (Applied Biosystems, Life Technologies Corp., Carlsbad, CA, USA). The primer sets used in this study were listed as follows: BMI1: BMI1-forward: 5′-AATCCCCACCTGATGTGTGT-3′; BMI1-reverse: 5′-GCTGGTCTCCAGGTAACGAA-3′; EZH2: EZH2-forward: 5′-GACTGGCGAAGAGCTGTTTT-3′; EZH2-reverse: 5′-TCTTTCGATGCCGACATACTT-3′; MPRL19 (internal control): MRPL19-forward: 5′-GGGATTTGCATTCAGAGATCAG-3’; MRPL19-reverse: 5′-GGAAGGGCATCTCGTAAG-3′.

### 4.5. Immunoprecipitation and ChIP Analysis

Five hundred micrograms (500 µg) of total cellular, cytoplasmic, or nuclear proteins were extracted and used for immunoprecipitation by adding 1 µg of anti-Hsp90α antibody in NP-40 cell lysis buffer and incubating at 4 °C for overnight. Ten microliters (10 µL) of Protein G Mag Sepharose Xtra beads (GE Healthcare Bio-Sciences, Pittsburgh, PA, USA) were then added and incubated at room temperature for 2 h. After washing steps, precipitated proteins were then eluted by adding 1× sample loading dye and incubated at 95 °C for 10 min. Immunoprecipitated proteins were analyzed by Western blot analysis. For ChIP analysis, cells were fixed with 1% formaldehyde at room temperature for 10 min. After quenching the formaldehyde with 125 mM glycine, the cells were lysed with mammalian cell lysis buffer (Pierce, Thermo Fisher Scientific, Inc., Waltham, MA USA) and chromatins were sonicated into fragments with lengths of 500 base-pairs. The ChIP analysis was then performed according to the previous report [40]. The c-Myc binding region of BMI1 promoter was detected by SYBR Green based quantitative PCR using the following primer set: forward primer: 5′-CACGGGCCTGACTACACCGACACT-3′; reverse primer: 5′-CACCGCTGAAGGCAGAGTGGAAAC-3′.

### 4.6. Determination of the Transcriptional Activity of c-Myc

Cells were seeded as 2 × 10^5^ cells/well in a six-well plate and co-transfected with pMyc-Luc reporter (Signosis, Inc. Santa Clara CA, USA) and pRL-TK Renilla reporter (Promega Corporation, Madison, WI, USA) plasmids at a ratio of 50:1 with TurboFect transfection reagent (Thermo Fisher Scientific Inc.) for 24 h. After treatment of 17-DMAG for a further 48 h, cells were lysed with 200 µL passive lysis buffer (Promega) and determined the luciferase activity with Beetle-Juice (for firefly luciferase (FLuc)) and Gaussia-Juice (for Renilla luciferase (RLuc)) substrates (PJK GmbH, Kleinblittersdorf, Germany), and luminescence was counted on a luminescence reader (Promega). The c-Myc transcriptional activity was determined according to the FLuc count after normalization to RLuc, which represented the transfection efficiency of each sample.

### 4.7. RNA Interference

The EZH2-specific shRNA carrying lentiviral vectors (TRCN0000040073, TRCN0000040076, and TRCN0000010475, obtained from the National RNAi Core Facility at the Institute of Molecular Biology, Academia Sinica, Taipei, Taiwan) were used to produce lentivirus particles as described in our previous report [40]. The knockdown of the EZH2 gene was performed with lentiviral transduction of mixed EZH2-specific shRNA virus particles as a ratio of 1:1:1 in the presence of 8 µg/mL polybrene and the successfully transduced cells were selected by 2 µg/mL puromycin (TOKU-E Co., Bellingham, WA, USA). A luciferase-specific shRNA (TRCN0000231722) was used as the negative control.

### 4.8. Overexpression of EZH2 with Lentivirus Transduction

EZH2 gene was amplified from cDNA of MDA-MB-231 cells with following primers: 5′-ATCGCTAGCATGGGCCAGACTGGGAAgAAATC-3′ and 5′-CGCATGCATTCAAGGGATTTCCATTTCTCTTTCG-3′. After digestion with NheI and NsiI, the amplified EZH2 fragment was cloned into pLAS5w.Pbsd-L-tRFP-C lentiviral vector (purchased form RNAi Core Facility in Academia Sinica, Taipei, Taiwan). The lentivirus particles were produced as described previously [24]. The transduction of lentivirus was performed by the addition of 8 µg/mL polybrene (Sigma) and blasticidin S (BSD, purchased from TOKU-E Co. Bellingham, WA, USA) was used for the selection of successfully-transduced cells at a concentration of 20 µg/mL.

### 4.9. Isolation of Cytosolic and Nuclear Proteins

The cytoplasmic and nuclear proteins of mammosphere cells were differentially isolated by FOCUS^TM^ Cytoplasmic and Nuclear Protein Fractionation Kit (G-Biosciences, St. Louis, MO, USA) according to the manufacture’s recommendation. Briefly, 1 × 10^6^ cells were harvested and washed with phosphate-balanced solution (PBS) and incubated in 20 µL ice cold SubCell buffer-I for 10 min. The cells were then lysed up and down ten times with a 20 G syringe needle. The cytoplasmic proteins were collected by adding 40 µL of 3× SubCell buffer-II and performing centrifugation at 700× *g* for 10 min. The nuclear pellet was further washed with 500 µL 1× SubCell buffer-II and SubCell buffer-III to remove the remaining cytoplasmic proteins. The nuclear proteins were finally obtained by lysing the nuclear pellet with 20 µL of SubCell buffer IV.

## 5. Conclusions

We discovered that 17-DMAG, an Hsp90 inhibitor, could inhibit the self-renewal of TNBC CSCs. This effect is associated with the suppression of BMI1 caused by the decreased amounts of Hsp90α-shuttled EZH2 and c-Myc to the nucleus. Our data suggested that 17-DMAG has potential applications as an effective anti-CSC chemotherapeutic drug.

## Abbreviations

17-DMAG	17-Dimethylaminoethylamino-17-demethoxygeldanamycin
ALDH	Aldehyde dehydrogenase
BCSCs	Breast cancer stem/progenitor cells
BMI1	B lymphoma Mo-MLV insertion region 1 homolog
EZH2	Enhancer of zeste homolog 2
Hsp	Heat shock protein
TNBC	Triple-negative breast cancer
